# Lord Lister: VI. Lister's Hypothesis of the Cause of Suppuration

**Published:** 1918-03-02

**Authors:** 


### LORD LISTER.

### VI.—Lister's Hypothesis of the Cause of Suppuration.

It was upon the hypothesis that not only hospital diseases, but suppuration also, is due to the action of something in the air that Lister originally founded his antiseptic system. The supposition that the infection of hospital diseases is conveyed through the air was common property. It was universally accepted and never questioned. The supposition that suppuration was similarly caused was Lister's own. It had never been suggested before, and, when he suggested it, was received partly with indifference, partly with scepticism. Not many paid any attention to the suggestion, and those who did waived it aside as unlikely and unimportant. No one but Lister himself recognised its vast significance. The conclusion to which he came, after years of careful observation, experiment, and meditation, was that something in the air produced decomposition or putrefaction—he used the terms interchangeably—of the discharges in the wound, and that the irritation of the decomposing discharges caused the wound to suppurate.

The two hypotheses, of the causation of hospital diseases and of the causation of suppuration, are quite distinct. The first was common property, and had prevailed for some time. The second was Lister's own, and it is the second that is of such profound importance. It is not too much to say that before Lister suggested this origin for suppuration, not only had no other origin been suggested, but the need for any suggestion was not recognised. Suppuration of wounds was looked upon as the natural and inevitable course of events, and it no more occurred to surgeons to ask themselves why wounds suppurate than it occurs to a gardener to ask himself why seeds germinate; or to a blacksmith to ask himself why iron, when heated, reddens and softens. In each case, the change was looked upon as part of the regular order of Nature. It was true that rare instances had been recorded in which wounds had healed by first intention, that is to say, without suppuration, but such instances were so rare that they attracted no attention. They were regarded as freaks of Nature for which there was no accounting. They were so rare that they formed in practice no exception to the rule that it is the nature of wounds to suppurate; and the notion that it might become possible to prevent wounds from suppurating had never occurred to anyone before Lister suggested it, and when he did suggest it, was looked upon as a mad whimsy, much as a blacksmith might regard the notion that a horseshoe could be heated in his forge and still remain black and hard.

The hypothesis that hospital diseases are due to the action either of the air itself, or of something in the air, upon the wound was common to all surgeons, but it never led any surgeon before Lister to make any attempt to exclude either the air itself or the infective matter that it was presumed to contain from gaining access to the wound. It was Lister who first drew this obvious practical inference. And he went much further. If, as he alone supposed, suppuration also was due to infection of the wound by something contained in the air, and carried by the air to the wound, then if this something could be prevented from gaining access to the wound, not only hospital diseases, but suppuration could be prevented, and wounds could be made to heal by first intention. This was Lister's reasoning. This was the great hypothesis upon which his system was founded. This was what he set himself to verify. It seems to us now very simple and very obvious, but it seems so to us because Lister has gone before and taught us. It was neither simple nor obvious to his contemporaries, and was scouted by them as in the highest degree chimerical.

The supposition that something that gains access to the wound is the cause of suppuration is the fruitful hypothesis at the foundation of Listerism, and has produced all the wonderful results that we see; but that the present generation, from want of familiarity with pre-Listerian surgery, is scarcely able to appreciate. The supposition that this something gained access to the wound *through the air alone* was the cause of much perplexity and of many failures, and retarded the triumph of Listerism for many years. To this point, that not only hospital diseases, but suppuration also, is due to the action of something that gains access to the wound through the air, Lister arrived by his own unaided observation and reasoning. From this point he would undoubtedly have gone on to reach the results that he ultimately did attain, but his progress would have been much slower, and his hypothesis would have rested upon a much less solid foundation, but that when he had reached this stage he became acquainted with the researches of Pasteur, which threw a flood of light upon the matter. Lister undoubtedly owed much to Pasteur, but his debt to Pasteur has been much exaggerated. It is commonly asserted, or if not asserted is implied, that Lister owed the whole of his hypothesis to Pasteur. The supposition displays gross ignorance of the history of the discovery. As has already been shown, Lister had achieved by far the greater and most important part of his discovery before ever he had heard of Pasteur and his researches. What he owed to Pasteur was the supposition, nay the certainty, that the something in the air that causes hospital diseases and suppuration is a living thing—a germ, as it was called. Lister had long decided that these processes in wounds are caused by the action of some foreign substance upon the wound; he had not decided on the nature of this foreign substance. He had decided that it is not the air itself, but something conveyed by the air;

Previous articles appeared Jan. 26, p. 351, Feb 2, p. 371, 9, p. 393, 16, p. 415, and 23, p. 435.

**LORD LISTER**—(*continued*).

he had not decided what it is that is conveyed by the air. Here it was that Pasteur helped him. From Pasteur's researches he divined that the foreign agent must be a living thing. This was what Lister owed to Pasteur, and it was much; but it was very far from all. Without Pasteur, Lister's discovery would have been incomplete; but it would still have been a great discovery. Pushed to its logical conclusion, it would probably have produced much the same results that we now see; but it would have taken much longer to produce them, and it is doubtful if they would ever have been as perfect.

The bearing of Pasteur's researches on Lister's practice was this, that they suggested to Lister that the infective material that he sought to exclude from wounds was living matter, and therefore could be excluded by guarding the wound with a barrier of some material that will destroy life in living matter. From thenceforward he set himself to discover such a material, and to apply it to wounds in such a way as to form an effective barrier against the entrance of living germs. This was the suggestion that Lister owed to Pasteur. This was the debt of Listerism to Pasteurism—this, no less and no more. To aggrandise one of these great men at the expense of the other is merely stupid. Both were great men. Each in his own department made great discoveries. Lister would still have made a great discovery even if he had never heard of Pasteur; but Pasteur's discoveries, or rather the application of them to his own speculations, an application that was itself a work of genius, immensely facilitated Lister's task by narrowing and defining the field of research.

Previous to the labours of Lavoisier at the end of the eighteenth century, the nature and cause of fermentation and putrefaction were as unknown and as little the subject of speculation as the nature and cause of suppuration. They were part of the order of Nature, like the burgeoning of trees in the spring, and the fall of the leaf in autumn, like the fall of rain and the rise of mist, and neither in the one case nor in the other did it occur to anyone to speculate as to cause or reason. From the time of Lavoisier, chemists busied themselves in seeking the nature and causes of putrefaction and fermentation. Though it was known that fermentation did not occur unless a ferment was present, and though it was discovered that the ferment is an organism, it was still supposed that the action was a purely chemical action, and that it mattered not whether the organism constituting the ferment was living or dead. Cagniard-Latour in 1837 showed for the first time that the ferment is not only an organic substance but a living organism, and cannot produce fermentation unless it is alive. Cagniard-Latour's conclusions were, however, scouted by Liebig, who was then regarded as an infallible oracle in chemistry, and the matter remained in obscurity until it was taken in hand by Pasteur.

Pasteur proved conclusively that Liebig was wrong, and that fermentation is due to the vital activity of a living ferment. He showed that different kinds of fermentation, the fermentation of grape-juice into wine, of wine into vinegar, of milk into lactic acid, of butter into butyric acid, are each due to the vital activity of a specific ferment, which can produce that particular variety of fermentation, and no other. He proved, moreover, that putrefaction is as much the work of micro-organisms as fermentation is, and this is where his work touched that of Lister, for it will be remembered that Lister regarded suppuration as due to the putrefaction of the discharges in the wound.

The most important bearing of Pasteur's researches upon Listerism were these three: First, that the micro-organisms to which fermentation and putrefaction are due are for practical purposes omnipresent. They float by millions in the air; they ride on particles of dust; they gather with the dust on surfaces and in crevices; they cling to solids; they are present in every hollow vessel; they are on the surface of every solid instrument and tool; they swarm on the surface layers of the earth; they are present at every depth in water and many other liquids, including even mercury; they are present in the bottles of the druggist; in the retort of the chemist; textile fabrics, even when they leave the loom, are full of them, and gather more with every day of use; every thread holds them, both on its surface and in its interior; and even the hands and the skin are impregnated with them.

The second of the great fundamental facts established by Pasteur is that in or on a given substance of limited size, and in a vessel of limited capacity the micro-organisms can be destroyed, so that a substance in which they have been destroyed, contained in a vessel in which they have been destroyed, will remain unfermented and unputrefied indefinitely.

The third fact of the utmost importance is that not only can a solid or liquid substance be sterilised, as it is now called, but the air also can be sterilised. By suitable means the micro-organisms can be filtered out of the air, and the air thus purified can be admitted into contact with a fermentible or putrescible substance that has been sterilised, and neither fermentation nor putrefaction will take place.

When it is remembered that Lister at this time attributed suppuration to the putrefaction of the discharges in a wound, the bearing of these discoveries of Pasteur on the genesis of suppuration is obvious, and Lister seized with avidity upon them.

(*To be continued.*)

